# Canine mesenchymal stem cells are effectively labeled with silica nanoparticles and unambiguously visualized in highly autofluorescent tissues

**DOI:** 10.1186/1746-6148-8-145

**Published:** 2012-08-27

**Authors:** Sei-Myoung Han, Hee-Woo Lee, Dong-Ha Bhang, Kyoung-Won Seo, Hwa-Young Youn

**Affiliations:** 1 Department of Veterinary Internal Medicine, College of Veterinary Medicine, Seoul National University, Seoul, 151-742, Korea; 2 Department of Veterinary Internal Medicine, College of Veterinary Medicine, Chungnam National University, Daejeon, 305-764, Korea

## Abstract

**Background:**

Development of a method for long-term labeling of cells is critical to elucidate transplanted cell fate and migration as well as the contribution to tissue regeneration. Silica nanoparticles have been recently developed and demonstrated to be biocompatible with a high labeling capacity. Thus, our study was designed to assess the suitability of silica nanoparticles for labeling canine mesenchymal stem cells (MSCs) and the fluorescence afficiency in highly autofluorescent tissue.

**Results:**

We examined the effect of silica nanoparticle labeling on stem cell morphology, viability and differentiation as compared with those of unlabeled control cells. After 4 h of incubation with silica nanoparticles, they were internalized by canine MSCs without a change in the morphology of cells compared with that of control cells. The viability and proliferation of MSCs labeled with silica nanoparticles were evaluated by a WST-1 assay and trypan blue exclusion. No effects on cell viability were observed, and the proliferation of canine MSCs was not inhibited during culture with silica nanoparticles. Furthermore, adipogenic and osteogenic differentiation of silica nanoparticle-labeled canine MSCs was at a similar level compared with that of unlabeled cells, indicating that silica nanoparticle labeling did not alter the differentiation capacity of canine MSCs. Silica nanoparticle-labeled canine MSCs were injected into the kidneys of BALB/c mice after celiotomy, and then the mice were sacrificed after 2 or 3 weeks. The localization of injected MSCs was closely examined in highly autofluorescent renal tissues. Histologically, canine MSCs were uniformly and completely labeled with silica nanoparticles, and were unambiguously imaged in histological sections.

**Conclusions:**

The results of the current study showed that silica nanoparticles are useful as an effective labeling marker for MSCs, which can elucidate the distribution and fate of transplanted MSCs.

## Background

Adult mesenchymal stem cells (MSCs) are a cellular tool with promising application in both veterinary and human medicine. MSCs have the potential to repair tissues [1-4] and improve the function of damaged organs. Thus, many clinical trials are in progress to evaluate the therapeutic potentials of MSCs for treating renal failure, diabetes mellitus and heart failure [5-9].

Tracking of stem cells is essential for evaluating cell replacement and therapeutic strategies [10]. In addition, long-term labeling of stem cells is critical to elucidate their fate, migration, and contribution to regenerating tissues. Conventional labeling methods used in cell and developmental biology include bromodeoxyuridine, chloromethyl dye, fluorescent in situ hybridization, 4'-6-diamidino-2-phenylindole (DAPI), and green fluorescent protein (GFP). However, these conventional labeling methods have limitations for use in labeling of stem cells [9,11]. Labeling methods using chemicals may affect stem cell functions [7], and most fail to fluoresce for a long period owing to photobleaching [12]. Moreover, secondary staining is sometimes needed for amplifying fluorescence to identify transplanted cells in tissue sections, and these protocols may produce false-positive results [7,13].

To overcome these issues, different kinds of nanomaterials have been created to track stem cells. Silica is a suitable nanomaterial for bifunctional or multifunctional cellular labeling because it is biocompatible, resistant to biodegradation in cellular environments and can be easily functionalized for bioconjugation [14,15]. Nanoparticles are coated with a shell of silica to avoid potential toxic effects. The silica shell contains luminescent organic dyes (ODs) such as rhodamine B isothiocyanate (RITC) or fluorescein isothiocyanate, resulting in a fluorescent and magnetic nanomaterial. In particular, incorporated ODs are more available than commercial OD products owing to their resistance to photobleaching. Because silica is stable under strong basic and acidic conditions, the fluorescent properties also remain stable [16]. In addition, many approaches involve endocytosis that contributes to higher uptake and retention of silica nanoparticles in MSCs [14,17]. However, no study has investigated the effects of silica nanoparticles as a labeling reagent for MSCs.

Therefore, we examined whether silica nanoparticles affected cell viability and the differentiation potential of MSCs, and evaluated the fluorescence efficiency in highly autofluorescent renal tissues.

## Results

### MSCs internalize silica nanoparticles and maintain typical MSC morphology

**Figure 1:**
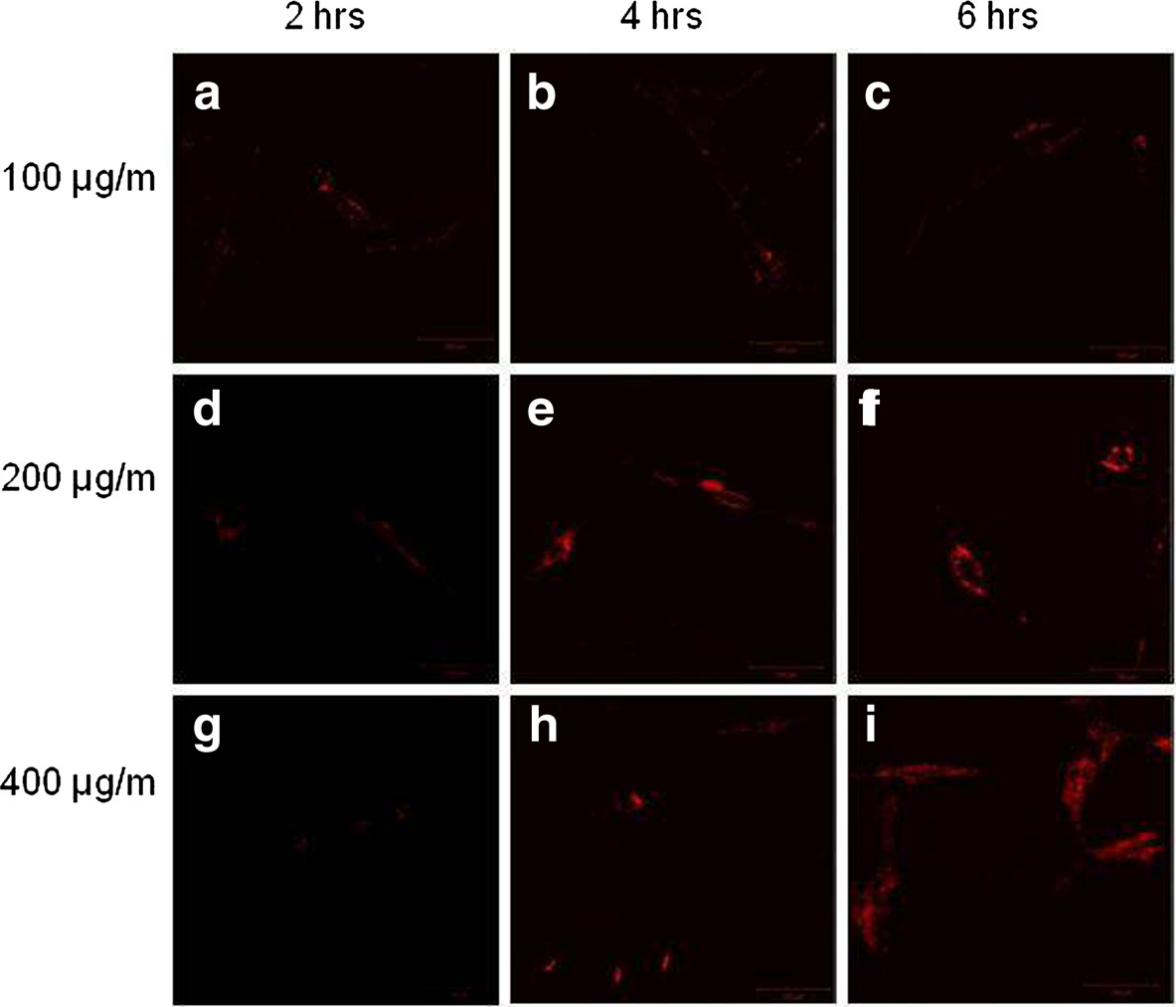
**Optimal concentration and incubation time of silica nanoparticles for labeling of MSCs.** UCBMSCs were incubated for more than 4 h with 200 or 400 μg/ml silica nanoparticles, which yielded visible fluorescent signals. However, no effective fluorescence occurred at 100 μg/ml. The majority of cells showed cytoplasmic fluorescence after 4 h of incubation. Magnification, ×400.

As shown in Figure 1, silica nanoparticles were taken up by cells, and concentrated fluorescence was observed inside canine MSCs with increasing incubation time. After 4 h of incubation, silica nanoparticles were visible in the cytoplasm of MSCs at labeling concentrations of 200 and 400 μg/ml. Using sufficient concentrations, silica nanoparticles were completely internalized by MSCs within 4 h (Figure 1). Thus, we used 200 μg/ml silica nanoparticles for subsequent experiments.

**Figure 2:**
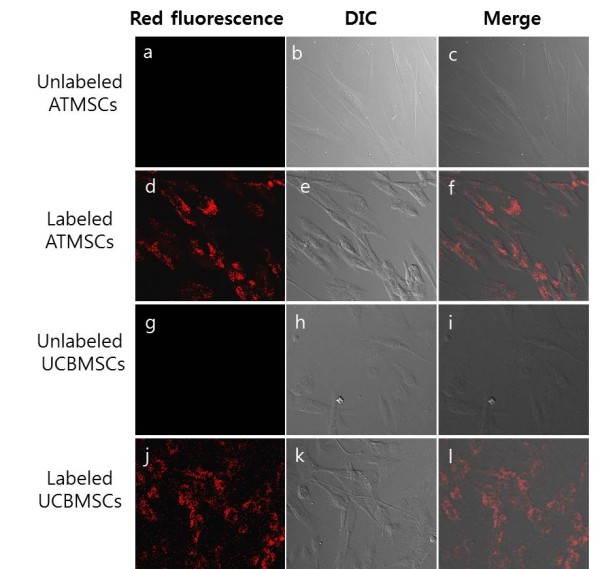
**Silica nanoparticles are internalized without affecting cell morphology.** Silica nanoparticles were internalized after 4 h of incubation with canine ATMSCs and UCBMSCs (c, h). Labeled canine MSCs (c, d) spread well on the dish, and showed fibroblastoid and spindle shapes. No notable differences in morphology were observed compared with that of unlabeled canine MSCs (a, b). Magnification, ×400.

The morphology of canine umbilical cord blood (UCB) MSCs and adipose tissue-derived (AT) MSCs was typical with fibroblastoid and spindle shapes, and the cells remained adherent (Figure 2b, h). After 4 h of incubation, silica nanoparticles were internalized by canine ATMSCs and UCBMSCs (Figure 2d, j) without altering the morphology of cells (Figure 2e, i), compared with that of control cells. The overlay (merged) images revealed co-localization of fluorescent cells in paired differential interference contrast (DIC) images (Figure 2c, f, i, l).

### Silica nanoparticles do not affect canine MSC viability or proliferation

**Figure 3:**
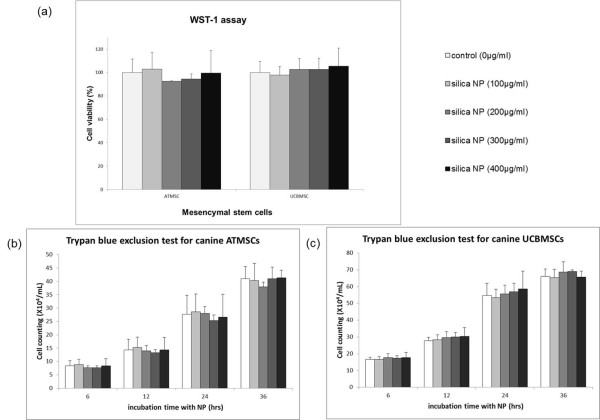
**Determination of cell viability and proliferation in the presence of silica nanoparticles for labeling of canine MSCs.** (**a**) Canine MSCs were incubated without (control) or with 100, 200, 300, or 400 μg/ml silica nanoparticles (NP) for 24 h, and then cell viability was measured by a WST-1 assay. No significant differences in metabolic activity were observed compared with that of the control during culture with silica nanoparticles. Each concentration was assayed in triplicate. (**b**, **c**) Trypan blue exclusion showed that the proliferation of canine MSCs was not inhibited. No significant differences in cell number were observed compared with that of the control during culture with silica nanoparticles. Each concentration was evaluated in triplicate. Data are the means ± standard deviations (P > 0.05, analysis of variance).

To evaluate potential cytotoxicity of silica nanoparticles in canine MSCs, a WST-1 assay, which measures cell viability relative to the metabolic activity, was performed after culturing the cells with the nanoparticles for 24 h. Figure 3a shows the effect of silica nanoparticles on the viability and metabolic activity of canine MSCs. The percentage of viable canine MSCs was not significantly different in the presence of increasing concentrations of silica nanoparticles, and was not significantly different from that of the control (P > 0.05, analysis of variance).

Because the WST-1 assay does not distinguish between the induction of cell death and the inhibition of proliferation, cell viability was measured by counting cells using trypan blue exclusion following exposure of the cells to silica nanoparticles. Silica nanoparticles at concentrations of 100, 200, 300 and 400 μg/ml had no significant effect on the cell viability of canine MSCs and their proliferation (Figure 3b, c). This result indicates that the presence of nanoparticles did not interfere with the viability or proliferation of canine MSCs.

### Silica nanoparticles do not inhibit the differentiation potential of canine MSCs

To assess the effect of silica nanoparticles on canine MSC differentiation, silica nanoparticle-labeled canine ATMSCs and UCBMSCs were cultured for 3 weeks under adipogenic and osteogenic induction conditions. Strong staining of Oil Red O or Alizarin Red S can interfere with the red fluorescence even in fluoroscope, we induced respectively for examination of Oil Red O or Alizarin Red S staining and red fluorescence.

**Figure 4:**
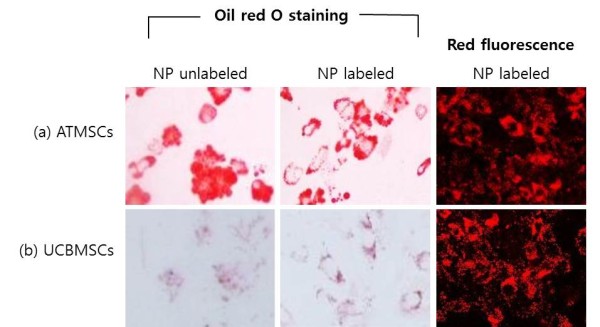
**Comparison of adipogenic differentiation between labeled and unlabeled canine MSCs.** UCBMSCs and ATMSCs were cultured in adipogenic differentiation medium for 21 days. Cells were then stained with oil red O. Red lipid droplets were visible in UCBMSCs and ATMSCs, indicating adipogenic differentiation. Adipogenic differentiation was not inhibited by silica nanoparticle labeling as compared with that of unlabeled cells. Magnification, ×200.

**Figure 5:**
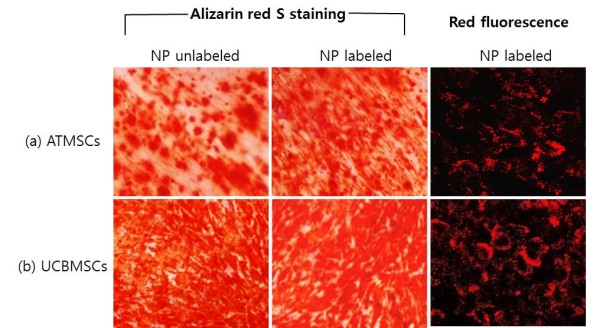
**Comparison of osteogenic differentiation between labeled and unlabeled canine MSCs.** UCBMSCs and ATMSCs were cultured in osteogenic differentiation medium for 21 days. Cells were then stained with alizarin red S. Red calcium deposition was observed following osteogenic induction. Osteogenic differentiation was not inhibited by silica nanoparticle labeling as compared with that in unlabeled cells. Magnification, ×200.

Adipogenic induction of canine MSCs labeled with silica nanoparticles resulted in expanded cell morphology. Staining with intracellular Oil Red O, a commonly used lipid dye (Figure 4), indicated that lipids had accumulated within the cells in small vacuoles, which appeared as droplets in canine ATMSCs and UCBMSCs. Adipogenic differentiation of silica nanoparticle-labeled canine ATMSCs and UCBMSCs was at a similar level as that of unlabeled cells. However, lipid accumulation in UCBMSCs was lower than that in ATMSCs, because of the canine UCBMSC characteristics [18]. When MSCs underwent osteogenic differentiation, they proliferated rapidly and formed tightly packed colonies. Cells were then stained with Alizarin Red S to assess mineralization (Figure 5). Osteogenic differentiation of canine ATMSCs and UCBMSCs labeled with nanoparticles occurred at a similar level as that of unlabeled cells. Using a fluoroscope, differentiated MSCs labeled with silica nanoparticles showed cytoplasmic retention of fluorescence (Figures 4 and 5). These results show that adipogenic and osteogenic differentiation of canine MSCs was not inhibited by silica nanoparticles.

### MSCs labeled with silica nanoparticles are unambiguously detected in renal tissue

**Figure 6:**
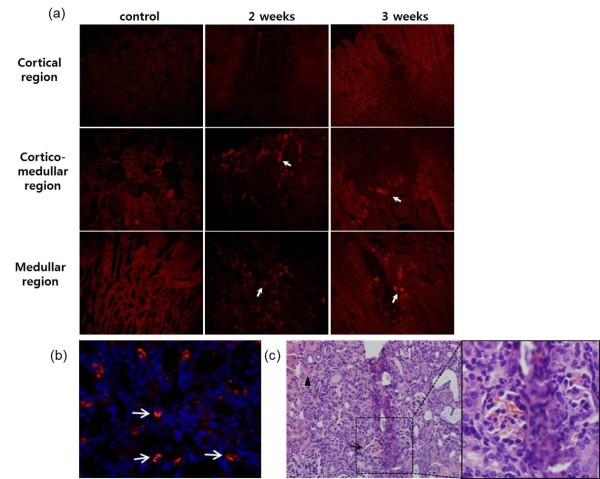
**Tracking of nanoparticle-labeled ATMSCs in the kidney.** (**a**) Transplanted silica nanoparticle-labeled ATMSCs were detected at the cortical medullary junction and in the medullary region at 2 and 3 weeks after injection. Fluorescent cells were not observed in the cortical region. Many fluorescent cells were unambiguously detected (arrows) in the corticomedullary and medullary regions. Cortical region: Magnification, ×100; corticomedullary and medullary region: Magnification, ×200. (**b**) ATMSC-injected kidneys were stained with DAPI and examined by confocal laser scanning microscopy. Arrowhead indicates co-localization of fluorescent silica nanoparticles and DAPI nuclear staining. Magnification, ×200. (**c**) The injection site was clearly observed in H&E-stained sections. Hemorrhages (black arrow) were detected and mild necrosis (black arrow head) was observed near the injection site. Magnification, ×100. The hemorrhagic region in (c) was magnified to show more detail. Magnification, ×400.

Approximately 2 × 10^5^ MSCs labeled with silica nanoparticles were injected into a mouse kidney after celiotomy. All treated mice remained healthy and gained weight normally. Mice were sacrificed after 2 or 3 weeks, and the gross appearance of the kidneys was normal except for mild hemorrhaging in the renal capsule at the injection site. Histological sections (4 μm thick) of renal tissue were prepared and examined for fluorescence. The renal cortex and medullary region were necrotic owing to the injection, which caused mechanical injury (Figure 6c). Silica nanoparticle-labeled MSCs were found predominantly in the corticomedullary and medullary regions that mainly consist of the proximal and distal tubules and the loop of Henle (Figure 6a). Co-localization of fluorescent silica nanoparticles and DAPI nuclear staining was observed (Figure 6b). This result indicates that silica nanoparticle-labeled MSCs were easily tracked, and silica nanoparticle fluorescence remained in canine MSCs for more than 3 weeks.

## Discussion

Our study demonstrated that canine MSCs can be effectively labeled by incubation with silica nanoparticles without impairing the cell morphology, viability or metabolic activity. In addition, silica nanoparticle-labeled MSCs exhibited a similar level of differentiation into adipocytes and osteocytes compared with that of unlabeled MSCs. Moreover, silica nanoparticle-labeled MSCs were uniformly visualized in the kidney at more than 3 weeks after transplantation.

These results demonstrate obvious benefits compared with other nanomaterials. Recently developed quantum dots show strong luminance and high photostability compared with those of conventional organic fluorophores [19]. However, the toxic potential of cadmium nanoparticles has remained a concern [20]. Boddington et al. reported [21] that the iron oxide nanoparticle ferucarbotran may impair MSC differentiation, which hinders their potential use as fluorescence tracers for stem cells. In contrast, the toxicological effects of amorphous silica have been characterized reasonably well, and this form has been found to be less toxic in animal experiments than that of other nanomaterials. Although some studies suggest that inhaling of materials such as colloidal silica by mice induces dose and size-dependent pulmonary effects [22,23], silica nanoparticles demonstrate no apparent toxicity in mice when injected via the tail vein [24]. Similarly, the safety of silica nanoparticles was confirmed in our study. The viability of unlabeled and silica nanoparticles-labeled cells, as measured by the WST-1 assay and trypan blue exclusion, was not significantly different, suggesting that silica nanoparticles are cytocompatible and do not induce cell death. Moreover, the differentiation capacity of MSCs toward multiple cell lineages was not affected by silica nanoparticle labeling. Labeled MSCs showed similar elaboration of mineralized matrix and accumulation of lipid droplets, compared with those of unlabeled MSCs during osteogenic and adipogenic differentiation, respectively.

We also demonstrated that silica nanoparticle-labeled MSCs can be histologically located in the kidney and tracked. Endocytosed silica nanoparticles persisted in expanded canine MSCs for up to 3 weeks. This 3 week period was sufficient for transplanted MSCs to migrate and differentiate in the host tissue. Furthermore, the fluorescence was not weakened until 3 weeks after transplantation. In contrast, superparamagnetic iron oxide nanoparticles show significant decreases in fluorescence after 1 month [25]. Such long-term labeling of stem cells will be beneficial for elucidating the relative contributions of native and engineered tissues to morphogenesis. Furthermore, silica nanoparticles can be imaged in renal tissue without additional staining. The kidney is a highly autofluorescent tissue owing to its extremely high metabolic rate, accumulation of flavins and lipofuscins, and vascularization [26]. Tissue autofluorescence has long posed a problem for studies of immunofluorescence labeling, and particularly for direct staining. Because it has been difficult to reduce autofluorescence in tissues, efficient labeling methods should be used to overcome this issue. We demonstrated that silica nanoparticles can be used as a reliable long-term tracking agent in autofluorescent tissues. Thus, we believe that this labeling method can track transplanted cells for extended periods in other autofluorescent tissues (e.g., liver, and pancreas).

We used an invasive method of injecting directly into the renal cortex after celiotomy to infuse stem cells into the kidney. After the abdominal incision surgery, the mice recovered immediately and gained weight normally, and no other complications were observed. Abdominal incisions can mobilize leukocytes and neutrophils to surgical areas, which is usually normalized within 7 days. In addition, we administrated broad-spectrum antibiotics to prevent infection from the surgery. Therefore, we believe that the effect of surgery on the mice and transplanted MSCs was insignificant. Renal tissues exhibited mechanical damage as shown by hemorrhaging and mild necrosis after the injection. The injury site may have been exposed to a number of inflammatory cytokines and chemokines owing to tissue inflammation. This microenvironment may be activated to enhance the homing of stem cells to these sites [27]. In the case of systemic MSC infusion, although some transplanted cells are located in the pathogenic area, most cells are trapped in the liver or enter systemic circulation [25]. In contrast, the detection rate of MSCs following intra-renal injection is much higher (20–50%) than that following systemic injection [28]. Thus, we chose direct renal injection to avoid systemic circulation of labeled MSCs, and most MSCs were observed in the corticomedullary junction and the medullary region at 2 or 3 weeks after injection (Figure 6). The medullary region consists of the proximal and distal tubules and the loop of Henle, in which the main functions involve regulation of water and electrolyte reabsorption. We presume that MSCs gradually moved to the damaged tubular regions and participated in tissue reconstitution. This relationship between MSC migration and their therapeutic effect needs further investigation. In any case, we expect that silica nanoparticle labeling will help elucidate the distribution and fate of transplanted MSCs.

Frangioni et al. [29] reported the following criteria to show that an ideal agent for tracking stem cells must (1) be biocompatible and safe; (2) not require any genetic modification or perturbation of the stem cells; (3) permit single cell detection in any anatomical location; (4) allow quantification of cell number; (5) have minimal or no dilution following cell division; (6) have minimal or no transfer to non-stem cells; (7) permit non-invasive imaging in the living subject over months to years; and (8) require no injectable contrast agent for visualization. Our study demonstrated that silica nanoparticles satisfied most of these criteria. However, we used only one cell line each for UCBMSCs and ATMSCs. Multiple cell lines will need to be tested with silica nanoparticles because of possible differences among cell lines. Based on our study, we suggest that silica nanoparticles represent a viable approach for labeling and tracking of MSCs.

## Conclusions

Our data demonstrate that silica nanoparticles uniformly and completely label canine MSCs in a biocompatible manner, resulting in unambiguous imaging with long-term persistence, even in highly autofluorescent tissues. Fluorescent silica nanoparticles are useful for labeling cells and may help identify the molecular mechanisms of MSCs, thereby contributing to the development of therapeutic methods.

## Methods

### Animals

BALB/c mice were purchased from Samtaco Bio Korea Co., Ltd. (Osan, Korea). Six-week-old male mice (27–33 g) were used in this study. All procedures involving mice were approved and followed the regulations of the Institutes of Laboratory Animals Resources (SNU-120523-2, Seoul National University, Seoul, Korea).

### Fluorescent silica nanoparticles

Fluorescent silica nanoparticles (NEO-STEM TSR50) were purchased from Biterials Co., Ltd. (Seoul, Korea). Silica nanoparticles were 50 nm in size and contained RITC conjugated to terminal silanol groups.

### Cell preparation

UCBMSCs were provided by the Adult Stem Cell Research Center of Seoul National University (Seoul, Korea). The isolation method and characterization of canine UCBMSCs have been described previously [18]. Canine ATMSCs were obtained from RNL Bio (Seoul, Korea). Both types of canine MSCs were cultured in low-glucose Dulbecco's Modified Eagle Medium (DMEM; Gibco, Grand Island, NY, USA) supplemented with 20% fetal bovine serum (FBS; Gibco) and maintained at 37°C with 5% CO_2_ in a humidified incubator. The medium was replaced every 3 days.

### Labeling of canine MSCs with silica nanoparticles

Passage 3 ATMSCs and UCBMSCs were cultured to 90% confluency. Then, fluorescent silica nanoparticles were added to the medium. To optimize the effective concentration and incubation time for labeling, canine MSCs were incubated for different time periods (2, 4 and 6 h) with various nanoparticle concentrations (100, 200 and 400 μg/ml). After incubation, the medium was replaced with fresh medium.

To prepare silica nanoparticle-labeled MSCs for transplantation, cells were incubated in nanoparticle-containing medium for 24 h. Cells (2 × 10^5^) were suspended in normal saline for injection into the kidney.

### Viability and proliferation of silica nanoparticle-labeled MSCs

Cell proliferation and viability were evaluated by a WST-1 assay and trypan blue exclusion. Cells were seeded in 96-well plates at 2 × 10^4^ cells/well. After 24 h, nanoparticles were added to the wells (0, 100, 200 or 400 μg/ml) to a final volume of 100 μl. Each concentration was assayed in triplicate, including the control wells. Cells were then incubated for 24 h with silica nanoparticles, and then 10 μl WST-1 reagent was added to each well. The reaction proceeded for 2 h at 37°C with 5% CO_2_. The absorbance of the samples at 450 nm was measured by a microplate reader. Cell viability (%) relative to control wells without nanoparticles was calculated by [absorbance]_test_ /[absorbance]_control_ × 100.

For trypan blue exclusion, canine UCBMSCs and ATMSCs were seeded into six-well culture plates and then incubated at 37°C with 5% CO2. After 24 h, MSCs were incubated with silica nanoparticles at 100, 200, 300 or 400 μg/ml for 6, 12, 24 and 36 h. Cells were then collected and stained with a 0.4% trypan blue solution. Enumeration of viable cells was carried out under a light microscope with a hemocytometer.

### Mesenchymal lineage differentiation assays of canine MSCs labeled with silica nanoparticles

Approximately 80% confluent canine MSCs at passage 2 in six-well plates were induced to differentiate into adipocytes or osteocytes for 21 days in adipogenic or osteogenic media, respectively.

MSCs were treated with adipogenic medium for 21 days to induce adipogenic differentiation. Adipogenic medium consisted of low-glucose DMEM containing 0.5 mM isobutyl methylxanthine (Sigma, St. Louis, MO, USA), 10 μM insulin (Sigma), 200 μM indomethacin (Sigma), 100 IU/ml penicillin, 100 μg/ml streptomycin and 20% FBS. The medium was changed three times a week. Oil red O staining was conducted to indentify lipid droplets.

MSCs were treated with osteogenic medium for 21 days to induce osteogenic differentiation. The osteogenic medium consisted of low-glucose DMEM containing 0.1 μM dexamethasone(Sigma), 10 mM β-glycerol phosphate (Sigma), 0.2 mM ascorbic acid (Sigma), 100 IU/ml penicillin, 100 μg/ml streptomycin and 20% FBS. The medium was changed three times a week. Alizarin red S staining (Sigma) was conducted to assess mineralized matrix content.

### Transplantation of silica nanoparticle-labeled canine MSCs into kidneys

We chose direct renal injection to determine whether silica nanoparticle-labeled canine MSCs could be easily detected in highly autofluorescent tissue. Male BALB/c mice were randomly divided into two groups, the normal saline-injected group (n = 3), and the nanoparticle-labeled canine MSC-injected group (n = 3). Under anesthesia following an intramuscular injection of Zoletile 50 (40 mg/kg, Virbac Laboratories, Carros, France), an abdominal incision was made, and the kidneys were exposed. ATMSCs were then injected into the kidneys. Cephalosporin (22 mg/kg) was injected intraperitoneally to prevent infection resulting from the surgery. Mice were sacrificed at 2 or 3 weeks after MSC transplantation.

### Tissue sample preparation

The kidneys were removed to examine the localization of injected MSCs. Kidney samples were embedded in OCT compound (Sakura Finetec USA Inc., Torrance, CA, USA) for cryosectioning (4 μm thick) and then examined by confocal laser scanning microscopy. Some specimens were stained with DAPI to visualize the nuclei. One MSC-injected kidney was 4% *(v/v)* formalin-fixed and paraffin-embedded, and then 4-μm-thick sections were processed for histology by hematoxylin and eosin (H&E) staining.

## Abbreviations

ATMSCs: Adipose tissue-derived mesenchymal stem cells; DAPI: 4'-6-diamidino-2-phenylindole; DMEM: Dulbecco's Modified Eagle Medium; FBS: Fetal bovine serum; MSCs: Mesenchymal stem cells; UCBMSCs: Umbilical cord blood mesenchymal stem cells.

## Competing interests

The authors declare that they have no competing interests.

## Authors' contributions

SMH designed the study, carried out the main study, assembled and analyzed the data, and drafted and wrote the manuscript. HWL contributed to study design and helped with data assembly and analysis, editing, and revision of the manuscript. DHB contributed to study design and was involved in assembling the data. KWS contributed to data interpretation and helped draft the manuscript. HWY contributed to study design and helped with editing and revision of the manuscript. All authors read and approved the final manuscript.
